# Portable Surface Plasmon Resonance Detector for COVID-19 Infection

**DOI:** 10.3390/s23083946

**Published:** 2023-04-13

**Authors:** Maciej Trzaskowski, Anna Mazurkiewicz-Pisarek, Jakub Waldemar Trzciński, Marcin Drozd, Rafał Podgórski, Anna Zabost, Ewa Augustynowicz-Kopeć

**Affiliations:** 1 Centre for Advanced Materials and Technologies CEZAMAT, Warsaw University of Technology, Poleczki 19, 02-822 Warsaw, Poland; 2 Faculty of Chemical and Process Engineering, Warsaw University of Technology, Waryńskiego 1, 00-645 Warsaw, Poland; 3 Department of Microbiology, Institute of Tuberculosis and Lung Diseases, Płocka 26, 01-138 Warsaw, Poland

**Keywords:** surface plasmon resonance, COVID-19, point-of-care detection, portable devices

## Abstract

Methods based on nucleic acid detection are currently the most commonly used technique in COVID-19 diagnostics. Although generally considered adequate, these methods are characterised by quite a long time-to-result and the necessity to prepare the material taken from the examined person—RNA isolation. For this reason, new detection methods are being sought, especially those characterised by the high speed of the analysis process from the moment of sampling to the result. Currently, serological methods of detecting antibodies against the virus in the patient’s blood plasma have attracted much attention. Although they are less precise in determining the current infection, such methods shorten the analysis time to several minutes, making it possible to consider them a promising method for screening tests in people with suspected infection. The described study investigated the feasibility of a surface plasmon resonance (SPR)-based detection system for on-site COVID-19 diagnostics. A simple-to-use portable device was proposed for the fast detection of anti-SARS-CoV-2 antibodies in human plasma. SARS-CoV-2-positive and -negative patient blood plasma samples were investigated and compared with the ELISA test. The receptor-binding domain (RBD) of spike protein from SARS-CoV-2 was selected as a binding molecule for the study. Then, the process of antibody detection using this peptide was examined under laboratory conditions on a commercially available SPR device. The portable device was prepared and tested on plasma samples from humans. The results were compared with those obtained in the same patients using the reference diagnostic method. The detection system is effective in the detection of anti-SARS-CoV-2 with the detection limit of 40 ng/mL. It was shown that it is a portable device that can correctly examine human plasma samples within a 10 min timeframe.

## 1. Introduction

COVID-19, caused by the coronavirus 2019-nCoV, is officially designated as a severe acute respiratory syndrome and represents a pandemic-level threat to global public health [1,2]. COVID-19 has turned into a worldwide health crisis, causing almost 759 million confirmed cases of disease and over 6.8 million deaths to date [3]. Coronaviruses (CoVs) are relatively large viruses (about 100 nm in diameter) containing a single-stranded positive-sense RNA genome encapsulated within a membrane envelope. The viral membrane is studded with glycoprotein spikes (S) that give coronaviruses their crown-like appearance. While coronaviruses infect certain types of animals other than humans, such as bats, animal hosts appear immune to coronavirus-induced illness [4]. The coronavirus SARS-CoV-2 genome encodes several structural proteins, including the glycosylated spike (S) protein, a major inducer of host immune responses. This S protein mediates host cell invasion by SARS-CoV-2 via binding to a receptor protein called angiotensin-converting enzyme 2 (ACE 2) located on the surface membrane of the host cells [5,6,7]. The SARS-CoV-2 particle is covered with a lipid membrane layer, and this membrane contains nucleocapsid protein [8]. According to the available information related to SARS-CoV epitopes in conjunction with in silico predictions, specific regions of SARS-CoV-2 encoding proteins have a high likelihood of being recognised by human immune responses. Analyses showed that T-cell epitopes were found to be predominantly associated with spike glycoprotein (S) and nucleoprotein (N), and B-cell epitopes with (S), (N), and membrane protein (M) [9,10,11].

Current mainstream diagnostic methods include nucleic acid PCR tests, direct viral antigen tests for detecting active infections, and indirect human antibody tests specific to SARS-CoV-2 to detect prior exposure. Nowadays, there are at least 420 nucleic acid tests, 179 immunoassay tests for antigens and 432 immunoassays for antibodies commercially available or under development for the diagnosis of COVID-19, according to the Foundation for Innovative New Diagnostics, a WHO collaborating centre for laboratory strengthening and diagnostic technology evaluation [12].

Antibody tests have some impressive advantages compared to molecular biology and antigen tests, although they do not confirm the presence of an active virus. An antibody test provides a much wider detection window. From a practical perspective, it is safer to collect blood samples than respiratory samples. Furthermore, the stability of human antibodies is much better compared to viral RNA during sample collection, fabrication, transport, and storage [13]. The lower detection limits of antibody tests are dictated mainly by a more uniform distribution of antibodies in blood than a virus in respiratory system samples, which often leads to false negative results in antigen and PCR tests [14]. Moreover, antibodies can successfully be detected in saliva in addition to blood [15] without requiring BSL-2 laboratories. Novel rapid antibody tests are being developed, confirming the thesis on the importance of such testing. For example, most recently, Ye et al. described a sensitive antibody test based on electrochemical impedance spectroscopy [16]. Rapid lateral flow tests for the presence of COVID-19 antibodies are now also available commercially from, e.g., Roche [17].

Regarding antibody types, IgM is considered an indicator of the early-stage infection, whereas IgG is an indicator of current or prior infection [18]. IgA and IgM can persist in the body for about 2 months, while IgG can last for more than three months [19,20]. To date, N and S proteins and their subunits have been used for antibody assay development. The sensitivity of the ELISA test for IgM against S was found to be significantly higher than that against N [21]. Both IgG and IgM against the receptor-binding domain (RBD) subunit were more sensitive than against N by ELISA [22]. Among the prokaryotically expressed recombinant N, N1, and N2 proteins, S1 and S-RBD-mFc showed the highest ELISA titres to detect IgM and IgG [23]. The same trend has been reported for eukaryotically expressed recombinant S1, S-RBD, and S-RBD-mFc spike proteins. The anti-N IgG in the magnetic-bead-based fluorescence immunoassay resulted in the highest sensitivity for detecting prior SARS-CoV-2 infection in saliva among the antigens such as ectodomain containing the S1 and S2 subunits, S1, S2, RBD, and N [15]. In a comprehensive study, N was more sensitive to a target than S and RBD for both IgG and IgM detection, while S was more sensitive than RBD and N for IgA detection. Additionally, cumulated data suggest that anti-S humoral responses were enriched among mild COVID-19 patients, whereas anti-N humoral responses were elevated in severe cases [24,25,26]. Therefore, the presented work relies on the RBD protein (SARS-CoV-2 Spike RBD Protein (Fc tag), expression system HEK293 cells) for antibody detection. 

The use of a surface plasmon resonance (SPR)-detecting platform for SARS-CoV-2 infections represents an alternative strategy for the fast on-line screening of large groups of patient blood samples, drawing on biological interaction detection without the need for developing sample signals, as the signal itself is created substantially in real time [27]. Successful demonstrations of the use of SPR in detecting SARS-CoV-2 have already been shown. Yano et al. investigated SPR sensing enhancement with gold nanoparticles. The results show the possibility of dropping the detection level of coronavirus N-protein to as low as 85 fM [28]. The group of Meng demonstrated a re-generable, high-throughput SPR system that detects antibodies against SARS-CoV-2 S1 protein in serum samples [29]. Djaileb showed a portable SPR apparatus able to detect anti-SARS-CoV-2 antibodies in samples of serum, plasma, and dried blood [30]. The SPR sensor Spreeta 2000 (S2k), because of its miniature size, simplicity of use, and robustness, has found applications in the construction of portable detection devices for on-site biological weapon detection [31] or the presence of tuberculosis bacteria in sputum, reported by our group [32]. Thus, we took an approach to use a similar portable system, based on S2k sensors, to detect anti-SARS-CoV-2 antibodies in human plasma.

In the presented work, we proposed a fast and highly automated antibody detection system for human plasma samples using a portable SPR device based on S2k multiple-use sensors. As a ligand molecule, which is responsible for binding antibodies in examined samples, we chose a specific region of spike glycoprotein encoding a receptor-binding domain of SARS-CoV-2, based on the literature research described above. The general idea of such a design is to perform antibody testing without the need of much laboratory equipment and highly qualified staff. From prior experience, we assumed that the detection procedure using such a device would not take more than 10 min. We also designed the system to be able to perform multiple analyses with a single sensing chip so it would be possible to use, for example, in point-of-care detection for a whole day without the need to change the sensor. Another advantage compared to other fast antibody-testing methods is a possible (but not demonstrated here) quantitative response obtained by the SPR system. Finally, the versatility of the presented platform enables us to perform other types of tests for COVID-19 infection, e.g., antigen testing from sputum, as has been proven before by our group. 

## 2. Materials and Methods

### 2.1. Materials

S2k sensor chips with gold surfaces modified with carboxylated polysaccharide were purchased from SensiQ Technologies, Inc., Oklahoma City, OK, USA. EDC, Sulfo-NHS, and other reagents for the immobilisation of proteins were supplied by Merck, Darmstadt, Germany. Anti-SARS-CoV-2 Spike Glycoprotein RBD antibody (ab277628) and recombinant human coronavirus SARS-CoV-2 Spike Glycoprotein RBD (ab273065) were purchased from Abcam, Cambridge, UK. Anti-SARS-CoV-2 QuantiVac ELISA test kits for the reference sample evaluation were purchased from Euroimmun, Wrocław, Poland. Reagents used to prepare buffers were purchased from Merck, Darmstadt, Germany and POCh, Gliwice, Poland. Plasma samples from both positive and negative COVID-19 patients were stored, prepared, and used for experiments in the microbiological laboratory of the National Institute of Tuberculosis and Lung Diseases in Warsaw, Poland.

### 2.2. Equipment

For the initial SPR experiments, MP-SPR Navi™ 220 NAALI with an auto-sampler and MP-SPR Navi Data Viewer software (version 6.4.0.5) (BioNavis, Tampere, Finland) was used. A SensiQ Discovery (SensiQ Technologies Inc., Oklahoma City, OK, USA) two-channel manual SPR platform designed to work with S2k chips was used to modify sensing chips by the chemical attachment of antibodies. The device was operated with the use of SensiQ Explorer software (ver. A.05), SensiQ Technologies Inc., Oklahoma City, OK, USA. An Ascor AP22 (Ascor Med Sp. Z o.o., Warsaw, Poland) double-syringe external pump was connected to the SPR platform. A prototype portable SPR detector for connecting and using S2k chips was designed and built. The design of the device has already been described elsewhere [31]. A miniature detector device comprising a microfluidic peristaltic pump, electromagnetic valve, and buffer and sample liquid transportation tubing was built. It can control the flow of liquid samples, buffers, and programme and read signals generated by S2k chips. The microfluidic system used in the device contained a flow cell made of polycarbonate capable of connecting a single S2k chip. The device was operated with the use of an external computer (laptop) with dedicated control and result analysis software. The device could be programmed to perform the automatic analytical procedure and send or display the overall results of the sample analysis.

### 2.3. Evaluation of Protein–Antibody System

A state-of-the-art multi-parameter SPR system (MP-SPR Navi™ 220 NAALI) with MP-SPR Navi Data Viewer software (version 6.4.0.5) was used to verify the capability of detecting anti-RBD antibodies using the viral protein. Firstly, gold sensing chips grafted with a layer of carboxyl groups were chemically modified by protein attachment. The chips were initially prepared with condition buffer: 100 mM NaOH + 2M NaCl; then, carboxylic groups were activated with the use of activation buffer: 50 mM MES/NaOH, pH 5.0; then, the solution of protein in the immobilisation buffer (5 mM MES/NaOH pH 5.0) was passed over the sensor surface for 20 min, followed by deactivation by a quenching buffer: 1M ethanolamine/HCl pH 8.5. These prepared chips were then used for the detection experiments. In the detection experiments, solutions of antibody (anti-SARS-CoV-2 RBD protein) in the running buffer: PBS pH 7.4 + 0.05% Tween 20 were prepared and passed over the surface of the sensing chips. After each concentration, regeneration buffer: 10 mM glycine/HCl pH 2.4 was passed to detach antibodies bound to the protein on the surface, followed by a short injection of running buffer to stabilise the experimental conditions before the injection of the next solution of antibody.

### 2.4. Preparation of S2k Sensing Chips

S2k chips pre-modified with a layer of carboxyl groups were modified by the attachment of RBD protein with the use of the same modification technique as in the case of BioNavis chips, using the same set of buffers. The modification was conducted with the use of the SensiQ Discovery SPR apparatus. The sensing channel of each S2k chip was coated with the protein, while the reference channel was left unmodified. Both channels were washed with a quenching buffer. These prepared sensors were used in the detection experiments with a prototype portable SPR detector.

### 2.5. Detection of Plasma Samples

**Figure 1:**
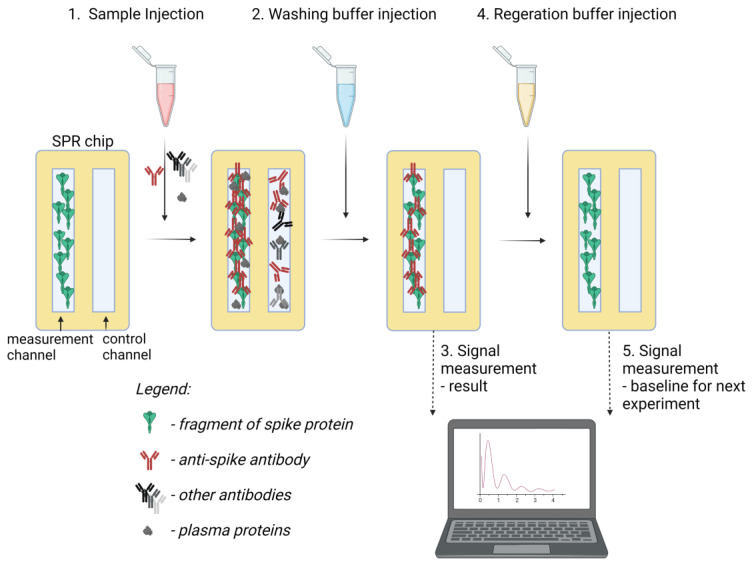
Graphical representation of the used test procedure for plasma sample measurement with SPR chip. Created with BioRender.com.

Plasma samples of patients (2 mL) were diluted 1:1 with a running buffer directly before the detection experiments. The prototype SPR detector was placed in the laminar-flow biosafety chamber. The procedure of the detection experiment was as follows (Figure 1): The tube for aspiring samples to the detector was immersed in the test tube containing the plasma sample (4 mL). The flow cell of the detector containing S2k was first washed with the running buffer (1 mL, 0.5 mL/min), and the resonance angle signal for both channels was measured (baseline measurement). Then, the sample was applied to the surface of the sensor (1 mL, sample flow 0.3 mL/min). Subsequently, the sensor was washed with a running buffer containing surfactant (Tween 20) to remove any non-specific binding (1 mL, buffer flow 0.5 mL/min), and the first sample signal was measured. Then, the chip surface was washed with a regeneration buffer (1 mL, buffer flow 0.5 mL/min) to detach antibodies from the protein coated surface. Next, a running buffer was passed over the chip (1 mL, buffer flow 0.5 mL/min) to condition the chip before the next sample. The second measurement of the signal was performed before the injection of the next sample after the stabilisation of the baseline due to the regeneration process. The procedure for single sample measurement lasted less than 10 min. The data analysis was performed by the baseline signal subtraction from the values of the signal collected from the measurement and from the control channels. Then, the signal from the control channel was deducted from the value of the measurement channel to obtain the measurement value. Each measurement point was carried out in 20 repetitions, and the mean result was calculated to exclude the influence of any noise caused by the environment or the detector itself. The obtained values were compared with the results of a reference test performed on the same samples (Euroimmun Anti-SARS-CoV-2 QuantiVac ELISA).

## 3. Results

### 3.1. Initial SPR Experiments

**Figure 2:**
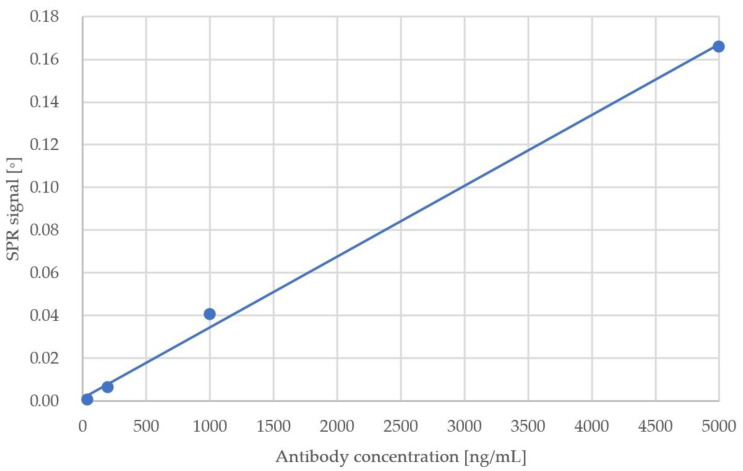
SPR signal against antibody concentration in the buffer.

Concentrations of antibodies ranging from 8 ng/mL to 5 µg/mL were tested versus a protein-modified SPR chip (Table 1, Figure 2). Both the immobilisation of RBD peptide and anti-RBD antibody detection were performed with the use of the MP-SPR Navi™ 220 NAALI system. The measurements were performed in a controlled environment in terms of temperature (22 °C), humidity (50%), and air quality (clean room laboratory).

### 3.2. Results of Plasma Sample Detection

**Figure 3:**
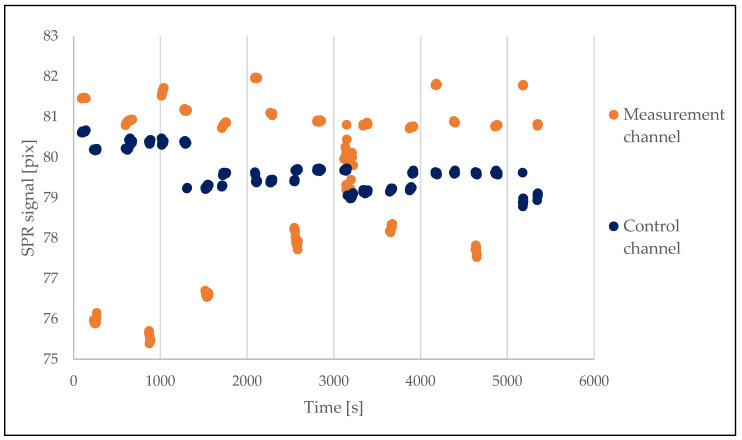
The SPR signal obtained during the experiment including 7 samples on a single chip. Raw data from measurement and control channels.

**Figure 4:**
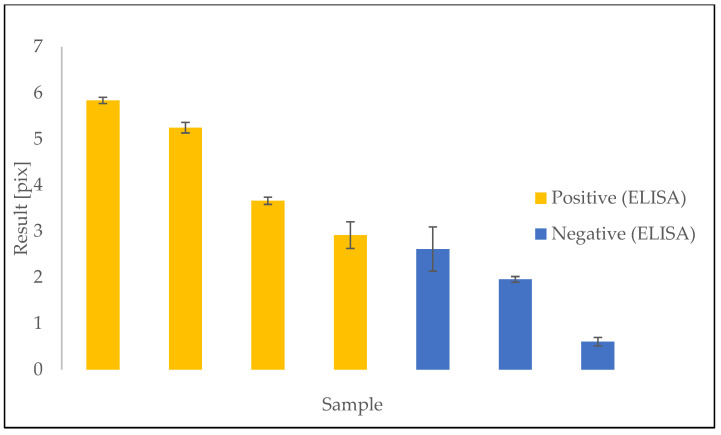
Results of the detection of seven plasma samples. Results compared with the reference according to the ELISA test.

Seven samples of plasma of both SARS-CoV-2-positive and -negative patients were investigated on a single chip. The results were calculated as described above, based on raw measurements (Figure 3) taken according to the scheme (Figure 1). The calculated results are shown below in Figure 4. The final time taken for the single detection procedure was below 10 min.

## 4. Discussion

The binding of antibodies in the initial SPR experiment resulted in a linear quantitative answer in the range of 40–5000 ng/mL, proving that the antigen–antibody system works with high sensitivity and precision and is capable of detecting anti-RBD protein in plasma. It also shows that there is a potential possibility to generate quantitative responses using the presented system.

We successfully identified all investigated samples with the use of the device, according to the reference ELISA method. However, samples of near threshold signal intensity gave similar result values.

Our results show the possibility of preparing a fast SPR method for the detection of COVID-19 infection able to be performed with a portable apparatus. It can also be potentially used in the detection of virus proteins in human sputum, as it has been previously demonstrated in our work that apparatus with a similar design could be used to examine such samples [29]. We must bear in mind, however, that the number of samples measured was insignificant. To validate such a test as an analytical method, a significant number of both SARS-CoV-2-positive and -negative patients should be tested to discover reliable result value thresholds.

The previous studies of SARS-CoV-2 epitope detection with the use of the SPR with a gold nanoparticle enhancement technique reported the detection limit of 4 pg/mL [28]. In other works, focused similarly to our research on qualitative analysis, the detection limit was either not specified [33] or reported very roughly as <8 µg/ mL [34]. The detection limit obtained by us (40 ng/mL) was not the lowest but was low enough for the quasi-online qualitative test, making the described method valuable in the terms of cost and applicability. A comparison between the hereby presented and some of the previously reported SPR systems for COVID-19 detection is shown in Table 2 below.

When compared with commercially available tests, the results obtained by our system are usually better in terms of detection time for a single sample (Table 3). Out of the producers that specify the time-to-result for a single test, only Lumigenex reports an analysis time similar to that obtained by our system [35]. Other producers specify their times-to-result most commonly as around 15 min [36,37,38,39]. However, there are also tests that take 18 [17], 22 [40], and even 30 min [41]. The advantage of the SPR system is the possibility to automatically record results of analyses. Additionally, our approach is better in terms of material usage, as the SPR sensing chips are of multiple use, compared to single-use lateral flow cassettes used by the cited systems.

The research on lateral flow tests for the detection of anti-SARS-CoV-2 antibodies is being continued. Such a construction of a test enables patients to potentially perform it by themselves [43]. The times to result in case of such tests are no shorter than 15 min. The anti-SARS-CoV-2 antibody detection system based on electrochemical impedance spectroscopy reported by Ye appears at least as good in terms of the time of analysis. The authors did not specify, however, the total time of sample analysis and focused on the sole measurement time, which is measured in milliseconds. It is also not specified whether a washing step to prevent unspecific interactions can be performed in this test. The chips used in this study are also disposable after a single use [16]. The microfluidic ELISA system reported by Tripathi seems to be an interesting and promising solution [44]. However, no data on detection parameters have been reported.

Our system, thanks to the use of S2k sensing chips based on a gold surface, is advantageous in terms of possible washing to remove any both specifically bound molecules as well as even removing the ligand and completely rebuild the binding layer. The system proposed by us can be easily changed in terms of the analyte detected by only changing the ligand immobilised on the sensor. It is thus potentially universal not only for the detection of other COVID-19 markers (e.g., antigens in sputum) but virtually any disease-correlated antibodies, antigens, or other markers.

Although the specificity of the detection was ensured by the use of protein fragments and washing the sensing chip with a buffer containing detergent, some negative samples also resulted in minor responses. This can be caused either or both by non-specific interactions with other proteins present in human plasma and insufficiently washed-out antibodies, which remain bound to the proteins on the sensor surface between procedures, causing control channel drift (see Supplementary Materials). The use of a stronger regeneration buffer should have ensured the evasion of the latter; however, stronger regeneration or longer washing times may be worth investigating. In this study, we focused on the quickest possible time-to-result. To provide better sensitivity, this parameter should be optimised.

## 5. Conclusions

The principle of the detection of anti-SARS-CoV-2 antibodies has been confirmed in laboratory conditions on state-of-the-art apparatus. The same principle has been used in a self-built prototype portable SPR detector. Real samples of plasma from patients have been examined. The results obtained matched those obtained with the reference method. The study showed that SPR can be a valuable technique for the fast point-of-care detection of COVID-19 infection. More data are required to improve the testing method—and more strictly, to define the positive–negative threshold and decrease the noise/unspecific binding that probably occurred during the experiments presented.

## Figures and Tables

**Table 1:** Results of initial anti-RBD antibody detection test.

Sample No.	Antibody Concentration (ng/mL)	SPR Response (°)
1	8	3.10 × 10^−5^
2	40	4.35 × 10^−4^
3	200	6.17 × 10^−3^
4	1000	4.05 × 10^−2^
5	5000	1.66 × 10^−1^

**Table 2:** Comparison of detection limits between present system and previously reported COVID-19 detection results obtained with SPR.

Reference	Detection Limit	Reported Time to Result (min)	Comments
Large gold nanoparticle-enhanced surface plasmon resonance [28]	4 pg/mL	Not specified	Sandwich assay
Multiplexed grating-coupled fluorescent plasmonics (GC-FP) biosensor platform [33]	Not specified	<30 min	-
Fibre optic surface plasmon resonance [34]	<8 µg/mL	30 min or 67 min depending on the mode	Two modes of operation: label-free and sandwich assay
Portable surface plasmon resonance detector	40 ng/mL	<10	-

**Table 3:** Comparison of detection time for single sample between present system and commercially available antibody detection kits for SARS-CoV-2.

Test Name	Producer	Reported Time to Result (min) ^1^
Elecsys^®^ Anti-SARS-CoV-2 [17]	Roche Diagnostics Corporation Indianapolis, IN, USA	18
BioCheck SARS-CoV-2 IgG and IgM Combo test [41]	BioCheck, Inc., South San Francisco, CA, USA	30
Cellex qSARS-CoV-2 IgG/IgM Rapid Test [36]	Cellex Inc., Cary NC, USA	15–20
SARS-CoV-2 IgG IgM Antibody Rapid Test Kit [35]	Lumigenex Co., Ltd., Suzhou, China	10
Novel Coronavirus 2019-nCoVAntibody Test [37]	Beijing Hotgen Biotech Co., Ltd., Beijing, China	15
SARS-CoV-2 Antibody Test [38]	Guangzhou Wondfo Biotech Co., Ltd., Guangzhou, China	15
Accre 6 [40]	Shenzhen Tisenc Medical Devices Co., Ltd., Shenzhen, China	22
Diagnostic Kit for IgM/IgGAntibody to Coronavirus (SARS-CoV-2) [39]	Zhuhai Livzon Diagnostics Inc., Zhuhai, China	15
Portable Surface Plasmon Resonance Detector	-	<10

^1^ Times to result of commercially available tests reported in the literature [42].

## Data Availability

The data presented in this study are available on request from the corresponding author.

## Supplementary Materials

The following supporting information can be downloaded at: https://www.mdpi.com/article/10.3390/s23083946/s1, Figure S1: Control channel signal drift over time.
