# Supplementary figures and images for: Portable Surface Plasmon Resonance Detector for COVID-19 Infection
Source: Sensors (Basel). 2023 Apr 13;23(8):3946. doi: 10.3390/s23083946 (PMC10144119; doi:10.3390/s23083946)

# Control channel drift over time

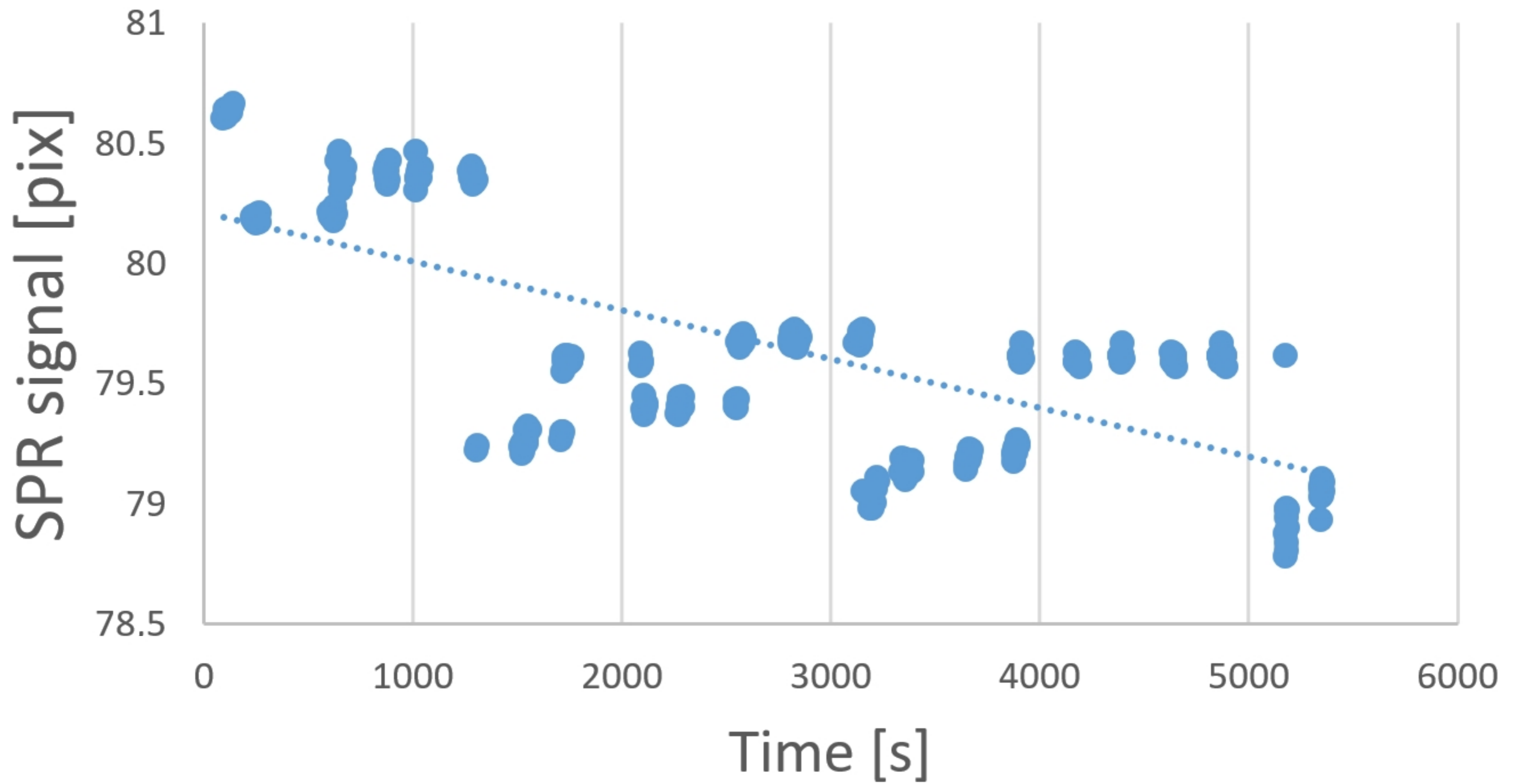

Supplement: Supplementary file 1 [file sensors-23-03946-s001.zip › sensors-2218453-supplementary.pdf]
